# RBM10 regulates alternative splicing of lncRNA Neat1 to inhibit the invasion and metastasis of NSCLC

**DOI:** 10.1186/s12935-022-02758-w

**Published:** 2022-11-05

**Authors:** Shan Cong, Xin Di, Ranwei Li, Yingshu Cao, Xin Jin, Chang Tian, Min Zhao, Ke Wang

**Affiliations:** 1 Department of Respiratory Medicine, the Second Hospital of Jilin University, No. 218, Ziqiang Street, Nanguan District, Changchun, 130041 Jilin Province China; 2 Department of Oncology and Hematology, the Second Hospital of Jilin University, Changchun, China; 3 Department of Urinary Surgery, the Second Hospital of Jilin University, Changchun, China

**Keywords:** NSCLC, RBM10, lncRNA Neat1, Alternative splicing, Invasion, Metastasis

## Abstract

**Background:**

Non-small cell lung cancer (NSCLC) accounts for more than 85% of the total cases with lung cancer. NSCLC is characterized by easy metastasis, which often spreads to bones, brains and livers. RNA-binding motif protein 10 (RBM10) is an alternative splicing (AS) regulator frequently mutated in NSCLC. We found that there were multiple peak binding sites between RBM10 and long non-coding RNA nuclear enriched abundant transcript 1 (LncRNA Neat1) by crosslinking-immunprecipitation and high-throughput sequencing (Clip-Seq). LncRNA Neat1 plays an indispensable role in promoting cancer in a variety of tumors and produces two splicing variants: Neat1_1 and Neat1_2. This study aims to explore the mechanism of RBM10 and LncRNA Neat1 in invasion and metastasis of NSCLC.

**Methods:**

Through histological and cytological experiments, we assessed the expression level of RBM10 protein expression. The interaction between RBM10 and Neat1 was evaluated via Clip-Seq and RNA immunoprecipitation assay. The effect of RBM10 on Neat1 and its splicing variants was identified by RT-qPCR. The effect of RBM10 and Neat1 on invasive and metastasis phenotypes of NSCLC was analyzed using transwell invasion assay and scratch test. Additionally, downstream signaling pathway of RBM10 were identified by immunofluorescence and western blot.

**Results:**

RBM10 exhibited low levels of expression in NSCLC tissues and cells. RBM10 inhibited the invasion and metastasis of NSCLC and recruited Neat1 and Neat1_2. Overexpression of RBM10 simultaneously inhibited Neat1 and Neat1_2, and promoted the expression of Neat1_1. On the other hand, silencing RBM10 promoted Neat1 and Neat1_2, and inhibited the expression of Neat1_1. From this, we concluded that RBM10 regulated AS of Neat1, and the tumor-promoting effect of Neat1 was mainly attributed to Neat1_2. RBM10 had a negative correlation with Neat1_2. In addition, RBM10 upregulated the expression of PTEN and downregulated the phosphorylation of PI3K/AKT/mTOR through Neat1_2, which ultimately inhibited the invasion and metastasis of NSCLC.

**Conclusion:**

The RBM10 regulated AS of Neat1 to cause the imbalance of Neat1_1 and Neat1_2, and RBM10 suppressed the activation of the PTEN/PI3K/AKT/mTOR signal by downregulating Neat1_2, finally affected the invasion and metastasis of NSCLC.

**Supplementary Information:**

The online version contains supplementary material available at 10.1186/s12935-022-02758-w.

## Background

Lung cancer is one of the most common malignant tumors globally, of which non-small cell lung cancer (NSCLC) accounts for approximately 85% [1]. Due to the high rate of invasion, metastasis, and postoperative recurrence, the 5-year overall survival rate of NSCLC patients is less than 15% [2]. A greater understanding of the molecular mechanisms underlying NSCLC cell invasion and metastasis is crucial to prevent tumor metastasis and improve survival.

RNA-binding proteins (RBPs), which are stably expressed in cells, bind to a variety of coding or non-coding RNAs (ncRNAs) and play an important role in the regulation of post-transcriptional gene expression, participating in splicing, processing, editing, methylation modification, and final decay [3]. RNA-binding motif protein 10 (RBM10) is a type of RBP located in the nucleus, which is frequently mutated in various types of cancers, especially lung adenocarcinoma (LA) [4]. RBM10 mutations were associated with tumor stage, lymphatic metastasis, and 5-year survival [5]. RBM10 inhibits the proliferation and invasion of lung cancer cells and promotes apoptosis by regulating tumor suppressor gene p53 [6] and Wnt/β-catenin signaling pathway [7]. RBM10 mutations are capable of changing the microenvironment of tumors and promoting their progression, rendering it a potential target for personalized cancer treatment [8].

Alternative splicing (AS) is an important mechanism by which cells regulate gene expression at the post-transcriptional level. Its products have a variety of spatial structures, which increase the diversity of transcriptomics. The expression levels of various splicing variants are also different, and their biological functions are also different. AS may have different or even opposing roles in tumor biologic behaviors, such as proliferation, apoptosis, angiogenesis, drug-resistance and metastasis [9]. RBM10 exerts a role in the regulation of AS similar to that of RBM5 [10]. NUMB is the most studied downstream effector of RBM10, and dysregulation of NUMB AS is frequently found in lung cancer [11]. RBM10 promotes the jump of NUMB exon 9, producing a NUMB isotype that leads to ubiquitination, and then the Notch receptor is degraded by the proteasome, thereby inhibiting the Notch signaling pathway to promote cell growth [12, 13]. To date, research on the AS function of RBM10 has mainly focused on coding RNAs. In the human genome, although nearly 75% of genes are transcribed into RNA, only about 2% of RNAs encode proteins; hence, the majority are ncRNAs [14]. Therefore, further research is necessary to clearly determine whether RBM10 affects ncRNAs via AS.

Nuclear enriched abundant transcript 1 (Neat1), a long non-coding RNA (LncRNA), exerts a cancer-promoting effect in some tumors, such as thyroid carcinoma [15], colon cancer [16], liver cancer [17], and melanoma [18]. Using crosslinking-immunprecipitation and high-throughput sequencing (Clip-Seq), we found that RBM10 showed multiple peak binding sites on LncRNA Neat1. Two Neat1 isoforms sharing the same 5’ end have been described, the constitutive short isoform (Neat1_1, 3.7 kb) and the stress-inducible long isoform (Neat1_2, 22.7 kb) [19]. Neat1_1 transcripts are processed by 3′ end processing complex CPSF6–NUDT21 and are cleaved at the polyadenylation signal located upstream. processing of Neat1_1 is inhibited by Hnrnpk, which binds to sequences between the CFIm binding site and the PAS [20, 21]. Alternative poly A is also regulated by Tardbp, which binds to the GU-rich motifs upstream of the PAS [22]. The 3′ end of Neat1_2 is cleaved by RNase P, and the non-polyadenylated transcripts are stabilized by triple-helix RNA structures found in the terminal region of Neat1_2 [23]. Neat1_2 is a major component of paraspeckles [24]. Therefore, it is one of our research directions to determine whether RBM10 regulates the AS of Neat1 to affect the occurrence and development of lung cancer.

In addition, PI3K/AKT/mTOR, as a classic signaling pathway in tumors, is often caused by abnormal PTEN gene function, resulting in inhibition of cell apoptosis, acceleration of cell cycle, promotion of angiogenesis and tumor invasion and metastasis [25–27]. The studies found that Neat1 can inhibit the expression level of PTEN in laryngeal cancer [28] and thyroid carcinoma [29] to promote malignant biological behavior of tumors. Previous research by our group also found that RBM10 can regulate RAP1/AKT/CREB to play a tumor suppressor role [30]. So whether RBM10 regulates the PTEN/PI3K/AKT/mTOR pathway through Neat1 is also one of the main objects of our research.

## Materials and methods

### Patients and samples

We collected 27 paired NSCLC and paracancerous tissues (> 3 cm away from tumor tissues) from patients who attended the Second Hospital of Jilin University between September 2019 and October 2021, including 14 cases of LA and 13 of squamous cell carcinoma (SCC). The patients did not receive chemotherapy or radiation before surgery. All patients were diagnosed according to the World Health Organization’s lung cancer criteria. This study was performed in accordance with the Declaration of Helsinki and was approved by the Ethics Committee of the Second Hospital of Jilin University (Changchun, China); all participants provided written informed consent. All tissue samples were assessed and confirmed by two pathologists independently, frozen in liquid nitrogen immediately after surgical resection, and then stored at − 80 °C until use.

### Cell culture and reagents

The human NSCLC cell lines (A549 and H1299), and human bronchial epithelial cell line (BEAS-2B) were obtained from Shanghai Genechem Co., Ltd. (Shanghai, China). A549 and H1299 cells were cultured in Roswell Park Memorial Institute Medium 1640 medium (Gibco, Carlsbad, CA, USA) containing 10% fetal bovine serum (FBS), and BEAS-2B cells were cultured in Dulbecco’s modified Eagle’s medium (Gibco, Carlsbad, CA, USA) containing 10% FBS.

### Cell transfection

The overexpression RBM10 lentivirus, silencing RBM10 lentivirus, and negative vector were constructed by Shanghai Genechem Co., Ltd (Shanghai, China). A549 and H1299 cells were cultured in six-well plates, pooled to reach 70%–80% confluency, and cleaned with phosphate buffer solution (PBS). Lentiviruses and negative vector were added to the cells according to the manufacturer’s instructions. After 24 h of lentivirus transfection, cells were screened in puromycin medium, and cell lines with stable overexpression and silencing of the RBM10 gene were established.

The Neat1_2 interference fragment was constructed by Suzhou GenePharma Co., Ltd. (Suzhou, China). A549 cells cultured in six-well plates were grown and pooled to reach 70%–80% confluency, and cleaned with PBS. The plasmid was added to the cell culture wells according to the instructions of Lipofectamine 2000 kit (Invitrogen, Carlsbad, CA, USA), and cultured for 24 and 48 h. The cells were used for subsequent experiments.

si-Neat1_2a: Forward 5′-GCUUCCACCCUGGAAGAUATT-3′

Reverse 5′- UAUCUUCCAGGGUGGAAGCTT-3′

si-Neat1_2b: Forward 5′-GCUAGUUUCCUUCCAGUUATT -3′

Reverse 5′-UAACUGGAAGGAAACUAGCTT-3′

### Western blot

After cells and tissues were fully lysed in radioimmunoprecipitation assay buffer supplemented with protease and phosphatase inhibitors (Beyotime, Jiangsu, China), protein concentrations were detected using a BCA protein detection kit (Beyotime, Jiangsu, China). Protein samples (30 µg) were separated by electrophoresis on 8%–15% sodium dodecyl sulfate-polyacrylamide gels and transferred to polyvinylidene difluoride membranes (#PIVH00010, Merck Millipore, Burlington, MA, USA). After blocking for 2 h, the membranes were incubated with specific primary antibodies (diluted in PBS containing Tween-20) at 4 °C overnight, followed by the respective secondary antibodies at room temperature for 1.5 h. Protein bands were detected using enhanced chemiluminescence detection kit (Merck Millipore) and quantified using Image J software (http://rsb.info.nih.gov/ij/, Bethesda, MD, USA). The used primary antibodies were anti-RBM10 (#14,423‐1‐AP, 1:500), GAPDH (#60,004–1-lg, 1:3000) from Proteintech Group (Chicago, IL, USA); anti-PTEN (#9188 T, 1:1000), anti-PI3K (#4257, 1:500), anti-p-PI3K (#abs130868, 1:500), anti-AKT (#4691, 1:800), anti-p-AKT (#4060, 1:500), anti-mTOR (#2983, 1:1000), anti-p-mTOR (#5536, 1:1000) from Cell Signaling Technology (Danvers, MA, USA).

### Reverse transcription quantitative polymerase chain reaction (RT-qPCR)

Total RNA was extracted from the tissues and cells using TRIzol reagent (Invitrogen, Carlsbad, CA, USA), and cDNA was obtained through reverse transcription using a kit (RR047A, Takara, Japan). RT-qPCR was performed using the SYBR Premix Ex TaqTM II (Perfect Real Time) kit (DRR081, Takara, Japan) on an ABI QuantStudio5 real-time PCR instrument (ABI, Foster City, CA, USA). Primers were synthesized by Shanghai Biotech Co., Ltd. (Shanghai, China) (Table 1). The Ct value of each well was recorded using β-actin as an internal reference. The 2^−ΔΔCt^ formula was used to calculate the relative expression.

**Table 1:** RNA primer sequence

RNA	Primer sequence
Neat1	Forward Primer 5′-CTTCCTCCCTTTAACTTATCCATTCAC-3′
	Reverse Primer 5′-CTCTTCCTCCACCATTACCAACAATAC-3′
Neat1_2	Forward Primer 5′-CTAGAGGCTCGCATTGTG TG-3′
	Reverse Primer 5′-GCCCACACGAAACCTTACAT-3′
β-actin	Forward Primer 5′-GTGGCCGAGGACTTTGATTG-3′
	Reverse Primer 5′-CCTGTAACAACGCATCTCATATT-3′

### Immunohistochemistry

A microtome was used to slice 4-μm sections of NSCLC and paracancerous paraffin-embedded specimens. The sections were stained with a Bond-Max automated immunohistochemical staining device (Leica, Germany). Image-Pro Plus analysis software (version 7.0; Media Cybernetics, Inc., Rockville, MD, USA) was employed to determine the color integral optical density (IOD) value of high-power field images (200 × , 400 ×) taken at random.

### Cellular immunofluorescence

A549 and H299 cells were seeded into confocal dishes at a density of 3 × 10^3^ cells/dish. When the cell density reached 60%, the cells were fixed for 20 min with 4% paraformaldehyde. Cells were stained using the Immunol Fluorescence Staining Kit (#E-IR-R321, Elabscience Biotechnology Co., Ltd., Wuhan, China); after blocking with 10% goat serum at 37 °C for 30 min, PTEN antibody (#9188 T, 1:1000) from Cell Signaling Technology (Danvers, MA, USA) was added and incubated at 4 °C overnight. Incubation with CY3 fluorescent secondary antibody (1:100) at 37 °C for 60 min in the dark followed. Images were taken using an inverted fluorescence microscope.

### Transwell invasion assay

Matrigel (#356,234, Corning Co., Ltd., Shanghai, China) was diluted with serum-free medium (1:8) and added to the upper part of the transwell chamber. The chamber was placed in a 24-well plate and incubated for 2 h at 37 °C in a humidified atmosphere containing 5% CO_2_. The cell concentration was adjusted to 10^5^ cells/100 μl, and 100 μl were added to the upper chamber, whilst 500 μl of serum-containing (15%) medium were added to the lower chamber and incubated for 24 h. The small chamber was removed, and the Matrigel and upper chamber cells were wiped off with a cotton swab and fixed with 4% paraformaldehyde for 10 min. The cells were stained with 0.1% crystal violet for 10 min. Fields of view were randomly selected under a high-power microscope, and Image J software (http://rsb.info.nih.gov/ij/, Bethesda, MD, USA) was used to count the number of penetrating cells.

### Scratch test

When the cell confluency reached approximately 90%, sterile pipette tips were used to apply a scratch to the middle of the well. The cells were cultured in serum-free medium for 24 h and cell migration was evaluated using Image J (http://rsb.info.nih.gov/ij/, Bethesda, MD, USA).

### Clip-Seq

Clip-seq is a revolutionary technology to reveal the interaction of RNA molecules with RBPs at the genome-wide level. Clip-Seq technology refers to the coupling of the treated cells or tissue samples under 254 nm ultraviolet light irradiation, and the RNA is captured by the specific antibody of RBP to form a protein-RNA complex. The RNA fragments protected by protein molecules in the complex are retained, and the remaining fragments are degraded to protect the target fragments. The ends of the RNA fragments are labeled with radioactive phosphates, so that the complex of the protein and the RNA fragments can be separated by denaturing SDS-PAGE gel, and then radioactive imaging can be performed. Proteinase K degrades the protein component of the protein-RNA complex, thereby preserving the RNA component, extracting RNA fragments, and then performing high-throughput sequencing (Additional file 1: Fig. S1). The Clip-seq experiment was jointly completed by our research group and Appreciate The Beauty of Life, lnc (Wuhan, China). CLIP-Seq data of RBM10 generated by GSE48066. As for protocols, please refer to references [31, 32].

### RNA immunoprecipitation (RIP)

A549 cells were collected at the logarithmic growth phase, and dissociated at 4 °C for 1 h. The prepared magnetic beads were incubated with the antibody mixture for 2 h, and the final product was purified. RIP was performed using a RIP Kit (#P0101; Guangzhou Geneseed Biotechnology Co., Ltd., Guangzhou, China) in accordance with the manufacturer’s instructions. The antibodies used were anti-RBM10 (#14,423–1-AP, 1:500) and a secondary IgG antibody (ab205718, 1:50) from Abcam (Cambridge, MA, USA).

### Statistical analysis

For all experimental results, statistical tests were performed using GraphPad Prism 6. Two-tailed Student’s t-tests were used for comparisons between the sample pairs. The correlation between RBM10 protein expression levels and LncRNA expression levels in tissues was examined using Pearson's Chi-squared test. Data are presented as mean ± standard deviation (SD), with a *P* value of < 0.05 indicating a statistically significant difference. All experiments were repeated at least three times.

## Results

### RBM10 expression is low in NSCLC

**Fig. 1:**
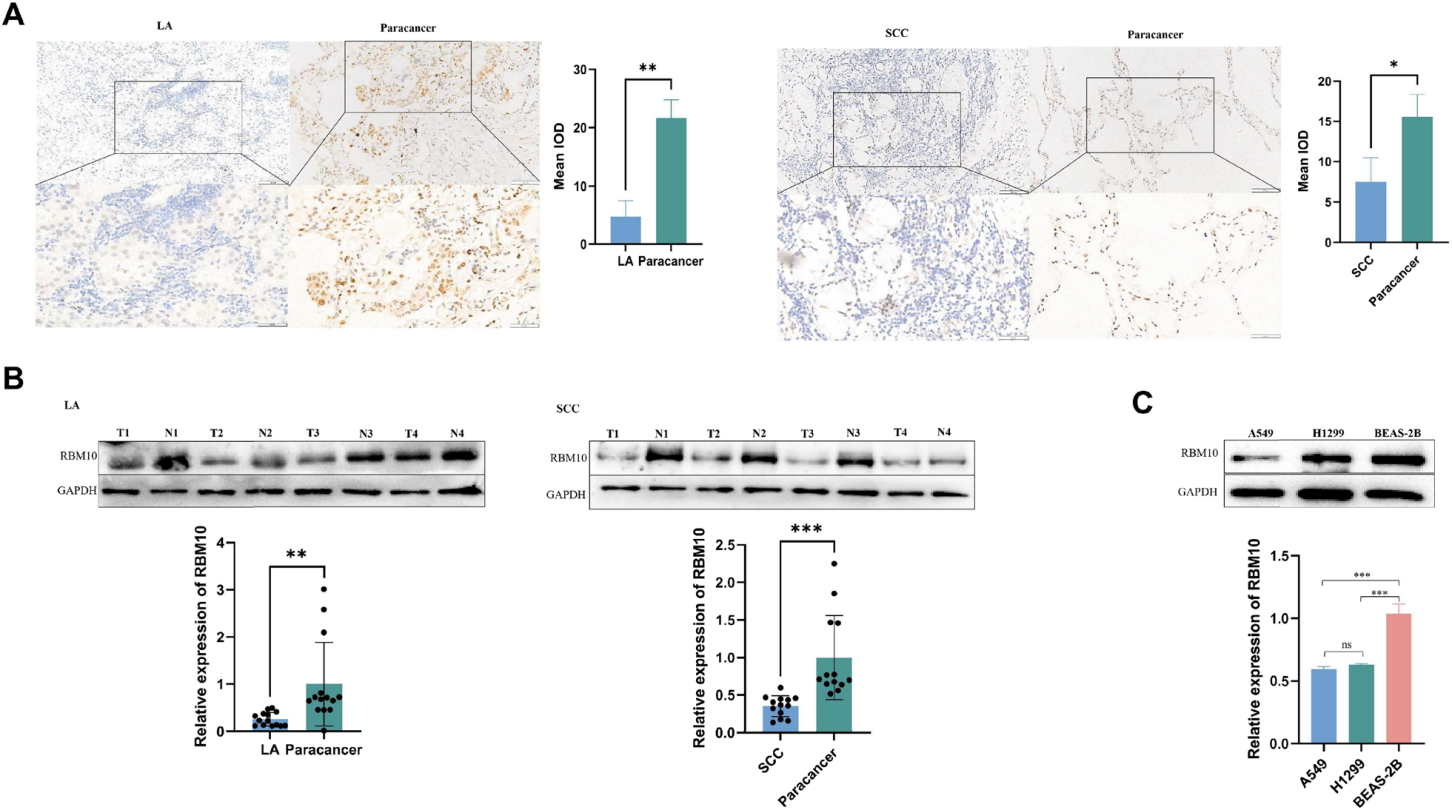
RBM10 expression is low in NSCLC tissue and cells. **A** Immunohistochemical detection of RBM10 protein expression in NSCLC and paracancerous tissues (200 ×, 400 ×). LA, lung adenocarcinoma tissue; SCC, squamous cell carcinoma tissue. **B** Western blot of RBM10 protein expression in representative tissue samples from NSCLC (T) and non-tumor specimens (N). **C** RBM10 protein expression in A549, H1299, and BEAS-2B cells. *p < 0.05, **p < 0.01, ***p < 0.001. Data are means ± standard deviation (SD)

We collected tissues from 27 patients with NSCLC, including cancerous and paracancerous tissues, 14 of which were LA tissues and 13 SCC tissues. We measured RBM10 protein expression using immunohistochemistry and western blot. The expression level of RBM10 in NSCLC tissues was lower than that in paracancerous tissues (Fig. 1A, B). Next, we compared the expression levels of RBM10 between NSCLC cell lines (A549 and H1299) and BEAS-2B cells, and found that the level was lower in A549 and H1299 cells compared to that in BEAS-2B cells (Fig. 1C).

### RBM10 overexpression suppresses cell invasion and metastasis, whereas RBM10 silencing enhances these processes in vitro

**Fig. 2:**
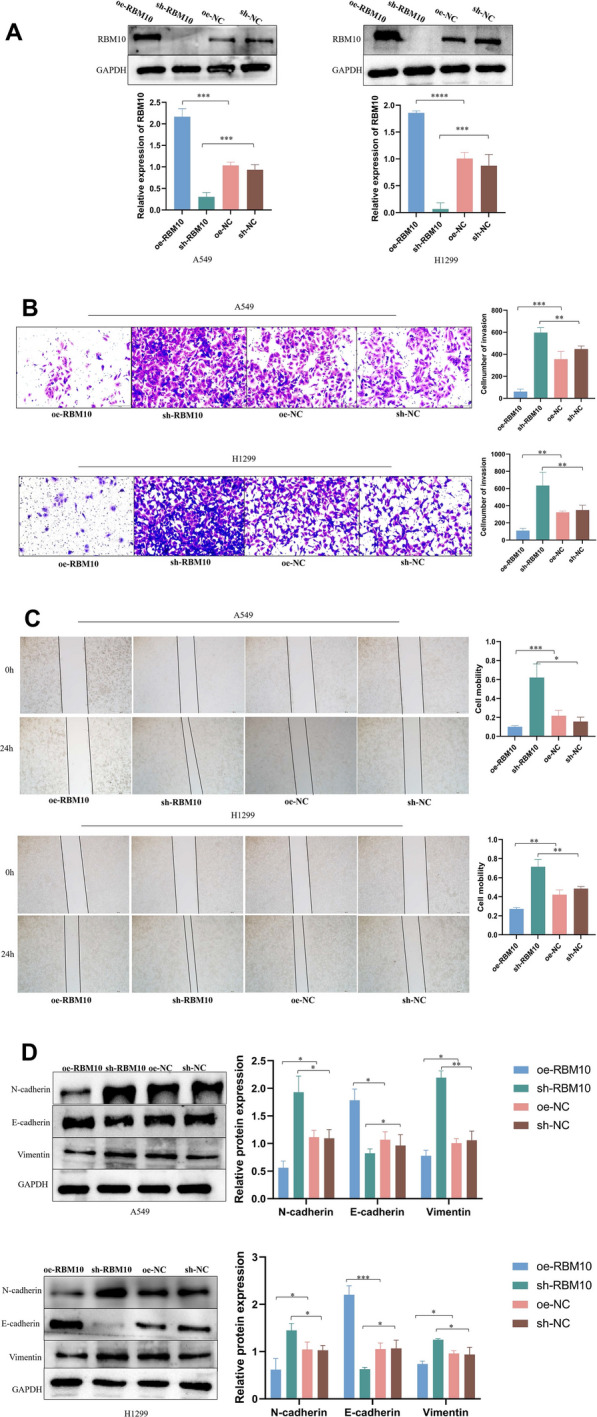
RBM10 inhibits invasion and metastasis of A549 and H1299 cells. **A** A549 and H1299 cells were transfected with lentiviruses expressing GV358-RBM10 (oe-RBM10), GV248-RBM10 (sh-RNA), and negative control (oe-NC and sh-NC). Using Western blot, total protein extracts were analyzed for RBM10 expression; negative control served as control respectively. **B** The effect of RBM10 on cell invasion was measured using the transwell invasion assay (200 ×). **C** The effect of RBM10 on cell metastasis was measured using the scratch test (40 ×). **D** The effect of RBM10 on the expression of N-cadherin, vimentin, and E-cadherin was measured using Western blotting analysis. *p < 0.05, **p < 0.01, ***p < 0.001, ****p < 0.0001. Data are means ± SD

We performed functional in vitro assays to explore the involvement of RBM10 in NSCLC progression. RBM10 was either overexpressed or silenced in A549 and H1299 cells through lentiviral transfection. Expression efficiency was determined by western blot (Fig. 2A). The results of the transwell invasion assay (Fig. 2B) and scratch test (Fig. 2C) indicated that RBM10 overexpression significantly attenuated cell invasion and metastasis, whereas silencing RBM10 markedly enhanced these processes. Epithelial-mesenchymal transition (EMT) is an important biological process in invasion and metastasis of tumor, and the upregulation of N-cadherin and Vimentin protein levels and downregulation of E-cadherin protein levels mark the occurrence of EMT. RBM10 overexpression decreased the expression of N-cadherin and Vimentin and increased the expression of E-cadherin. In contrast, RBM10 silencing reversely regulated these proteins (Fig. 2D).

### RBM10 promotes PTEN expression and inhibits the phosphorylation of PI3K/AKT/mTOR signaling pathway

**Fig. 3:**
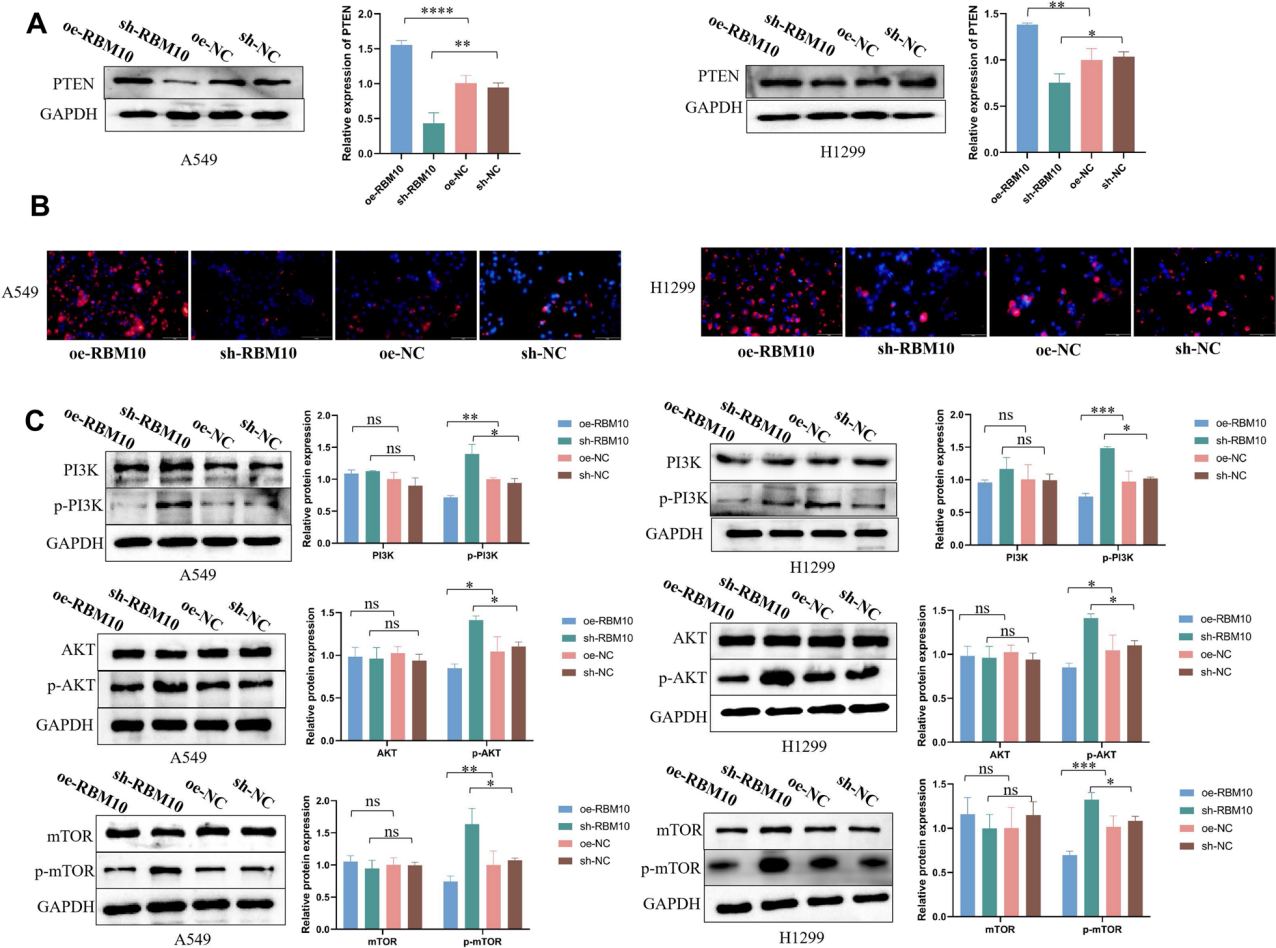
RBM10 promotes PTEN expression and inhibits the phosphorylation of PI3K/AKT/mTOR signaling pathway. **A** PTEN protein expression in oe-RBM10 and sh-RBM10 NSCLC cells by Western blot analysis, normalized to GAPDH. **B** Immunofluorescence qualitative analysis of PTEN protein expression (100 ×). The blue fluorescence represents the nucleus, the red fluorescence represents the PTEN protein, and the red fluorescence is mainly located in the cytoplasm. **C** The phosphorylation of PI3K, AKT, and mTOR in oe-RBM10 and sh-RBM10 NSCLC cells by Western blotting analysis, normalized to GAPDH. *p < 0.05, **p < 0.01, ***p < 0.001, ****p < 0.0001. Data are means ± SD

Overexpression of RBM10 resulted increased the PTEN expression and decreased phospho‐PI3K, phospho‐AKT, and phospho-mTOR levels in NSCLC cells; in contrast, the PTEN expression was decreased and the phosphorylation of PI3K/AKT/mTOR was greatly increased in RBM10‐silenced NSCLC cells compared to the control group (Fig. 3A–C).

### RBM10 regulates the AS of Neat1

**Fig. 4:**
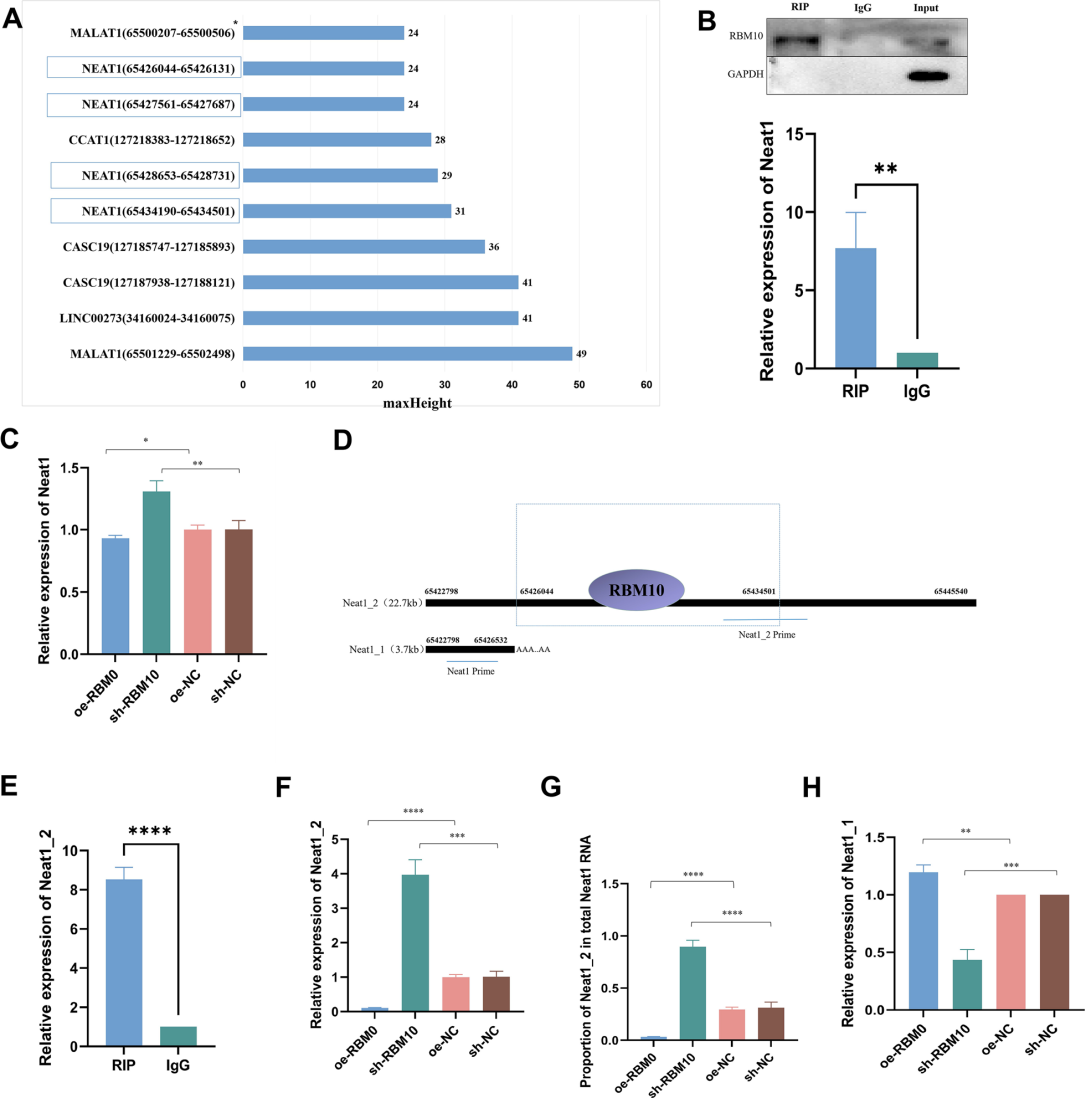
RBM10 regulates Neat1 AS. **A** Clip-seq analysis revealed the top 10 LncRNA binding sites in the RBM10 binding peak according to maxHeight. **B** RIP assay to verify the binding between RBM10 and Neat1. **C** Neat1 expression in A549 cells after RBM10 overexpressing and silencing by RT-qPCR, normalized to β-actin. **D** Structure of NEAT1_1 and Neat1_2 and binding site of RBM10 to Neat1. **E** RIP assay to verify the binding between RBM10 and Neat1_2. **F** Neat1_2 expression in A549 cells after RBM10 overexpressing and silencing by RT-qPCR, normalized to β-actin. **G** Proportion of Neat_2 in total Neat1 after RBM10 overexpressing and silencing. **H**. Neat1_1 expression in A549 cells after RBM10 overexpressing and silencing. *p < 0.05, **p < 0.01, ***p < 0.001, ****p < 0.0001. Data are means ± SD

Clip-seq analysis showed that RBM10 could bind 353 lncRNAs, and among the top 10 maxHeight binding peaks, RBM10 had four binding sites with Neat1 (Fig. 4A). We confirmed the binding between RBM10 and Neat1 by RIP experiment (Fig. 4B). The expression level of Neat1 decreased after RBM10 overexpression, but increased after RBM10 silencing (Fig. 4C). We further explored the structure of Neat1 and its transcripts, and found that the main binding site of RBM10 was located at 65,426,044–65,434,501, and the binding site was mainly concentrated on NEAT1_2 (Fig. 4D). We confirmed that RBM10 could recruit Neat1_2 through RIP again (Fig. 4E). Moreover, we found that RBM10 overexpression inhibited Neat1_2 expression, while RBM10 silencing promoted Neat1_2 expression (Fig. 4F). In addition, the proportion of Neat1_2 in Neat1 decreased significantly in RBM10 overexpressed A549, while the proportion of Neat1_2 in Neat1 increased significantly after RBM10 silencing (Fig. 4G). Because Neat1_1 has no specific primer, based on the ratio of Neat1_2 in Neat1 and the expression of Neat1 and Neat1_2 we concluded that Neat1_1 increased after RBM10 overexpression and decreased after RBM10 silencing (Fig. 4H). Together, these results suggest that RBM10 regulates the balance between the two splicing variants of Neat1 through AS.

### The expression of Neat1_2 increases in NSCLC cells and tissues, and RBM10 has a negative correlation with Neat1_2

**Fig. 5:**
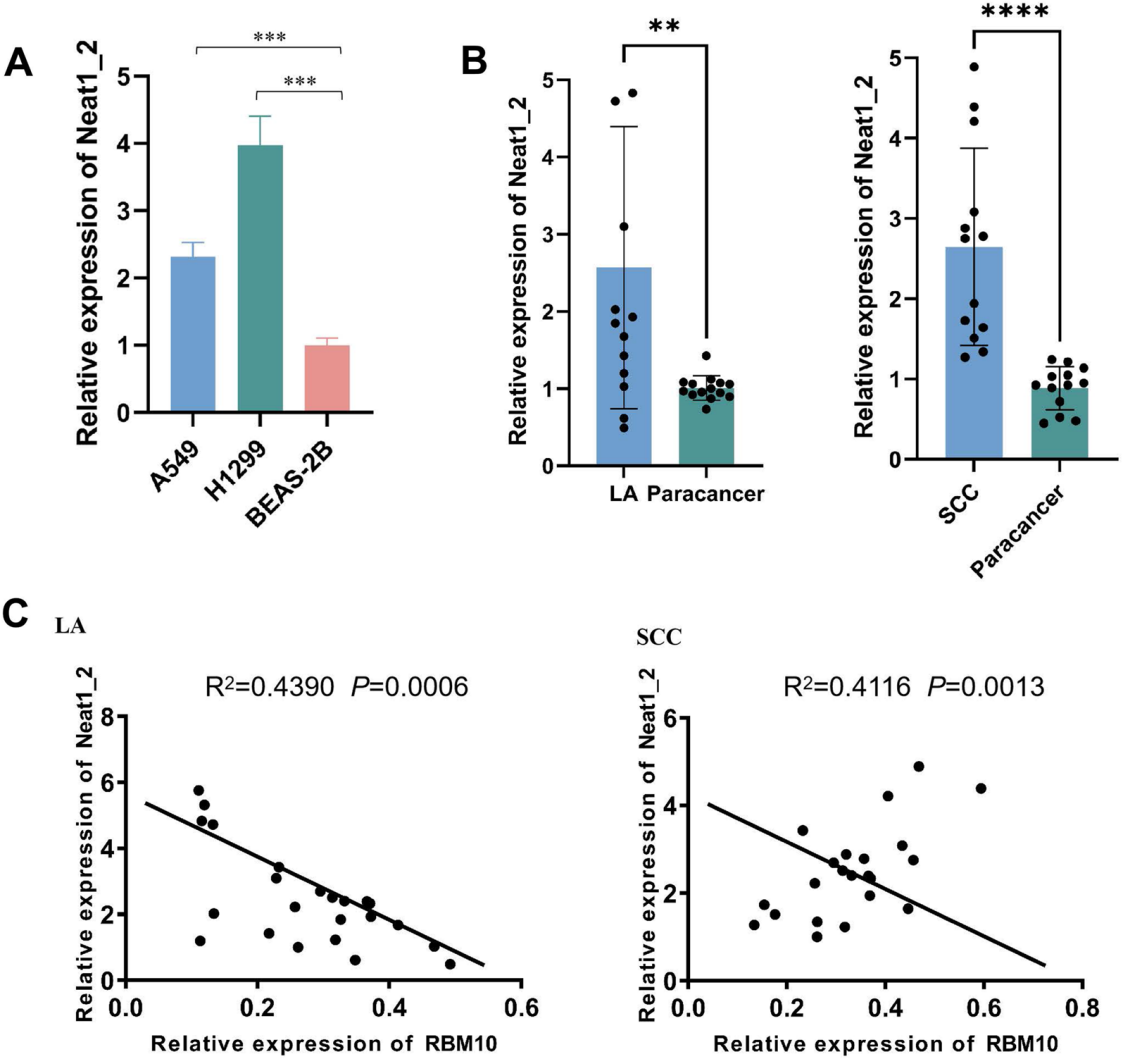
The expression of Neat1_2 increased in NSCLC cells and tissues, and RBM10 is clinically correlated with Neat1_2. **A** Neat1_2 expression in NSCLC cell lines and HBE cells by RT-qPCR, normalized to β-actin. **B** Expression of Neat1 in NSCLC and paracancerous tissues as determined using RT-qPCR. LA, lung adenocarcinoma tissue (n = 14); SCC, squamous cell carcinoma tissue (n = 13). **C** Pearson correlation of RBM10 and Neat1_2 expression in LA and SCC. **p < 0.01, ***p < 0.001, ****p < 0.0001. Data are means ± SD

By RT-qPCR, the results showed that Neat1_2 expression was significantly upregulated in NSCLC cell lines compared to that in BEAS-2B cells (Fig. 5A). Furthermore, compared with the paracancerous tissue group, Neat1_2 expression was significantly higher in the NSCLC group on RT-qPCR analysis (Fig. 5B). Pearson correlation analysis indicated a negative relationship between RBM10 and Neat1 in LA tissues (R^2^ = 0.4390, *p* < 0.05) and SCC tissues (R^2^ = 0.4116, *p* < 0.05) (Fig. 5C).

### RBM10 inhibits the invasion and metastasis potentials of NSCLC cells via Neat1_2

**Fig. 6:**
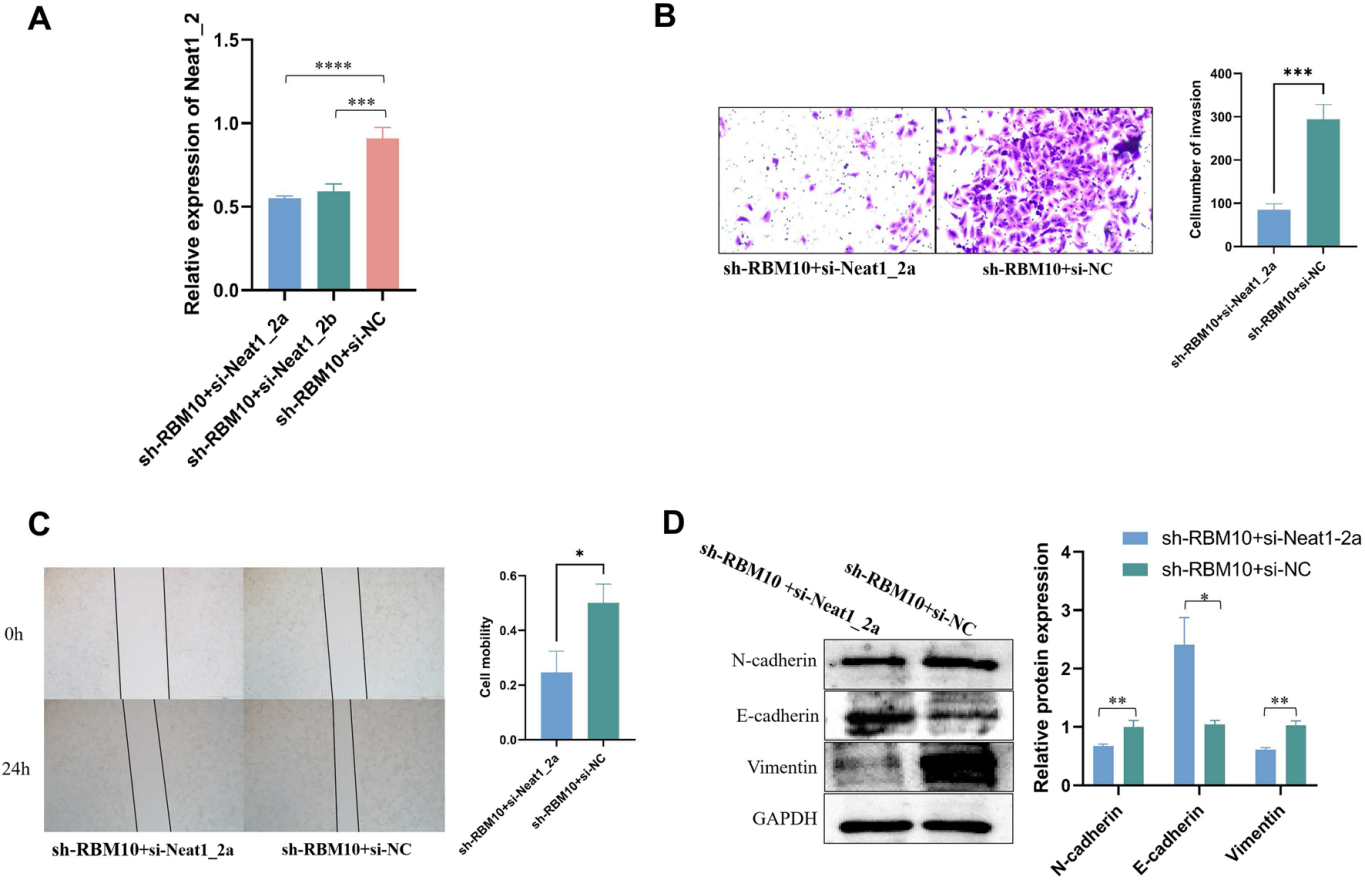
RBM10 inhibits the invasive and metastasis potentials of NSCLC cells via Neat1_2. **A** Expression of Neat1_2 in response to Neat1 silencing using different siRNAs as determined by RT-qPCR, normalized to β-actin. **B** Cell invasion was measured using the transwell assay (200 ×). **C** Cell metastasis was measured using the scratch test (40 ×). **D** EMT related proteins were measured using Western blotting analysis. *p < 0.05, **p < 0.01, ***p < 0.001, ****p < 0.0001. Data are means ± SD

We stably silenced Neat1_2 in RBM10-silenced A549 cells (Fig. 6A). The results of the transwell invasion assay (Fig. 6B), scratch test (Fig. 6C), and the expression of EMT relative proteins (Fig. 6D) illustrated that silencing of Neat1_2 reversed the impact of sh-RBM10 on cell invasion and metastasis. These results indicate that an inhibition of RBM10 promotes the invasion and metastasis of NSCLC cells by promoting the expression of Neat1_2.

### RBM10 regulates PTEN/PI3K/AKT/mTOR signaling pathway via Neat1_2 in NSCLC cells

**Fig. 7:**
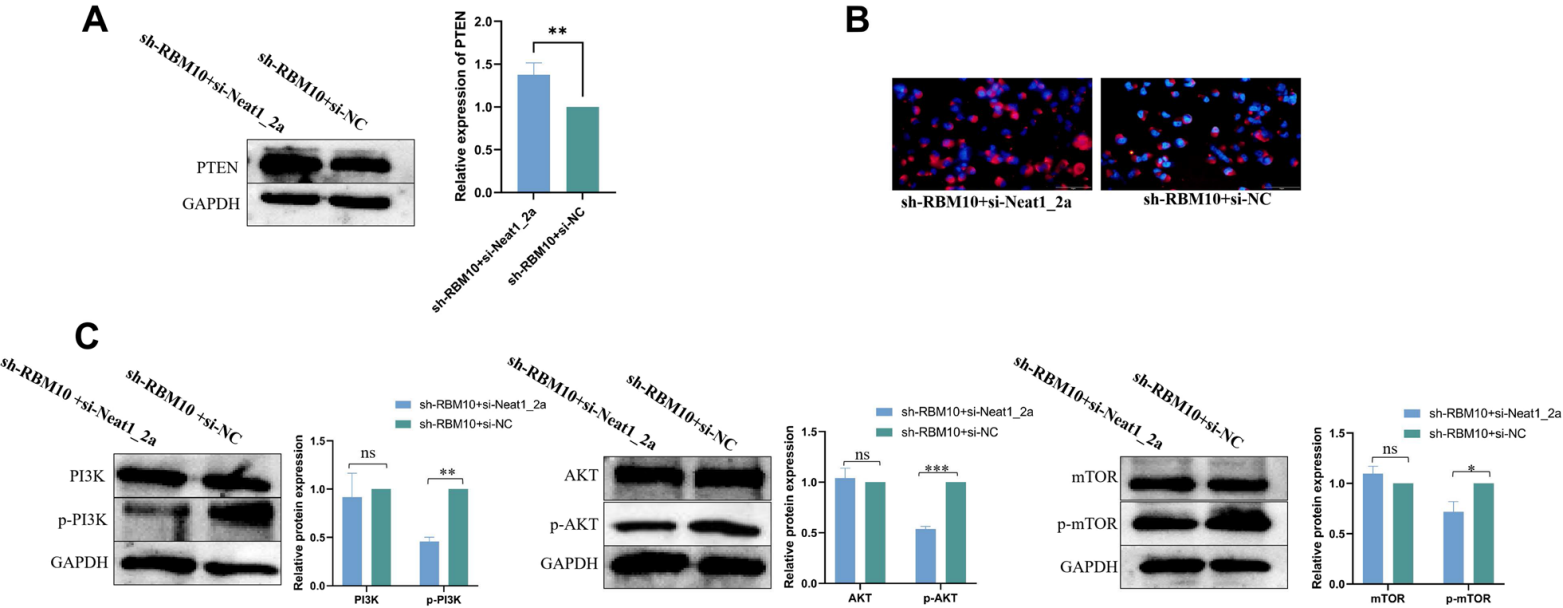
RBM10 regulated PTEN/PI3K/AKT/mTOR signaling pathway via Neat1_2 in NSCLC cells. **A** PTEN protein expression after silencing Neat1 in RBM10-silenced A549 cells by Western blot analysis, normalized to GAPDH. **B** PTEN protein expression after silencing Neat1 in RBM10-silenced A549 cells by cell immunofluorescence assay. **C** The phosphorylation of PI3K, AKT, and mTOR after silencing Neat1 in RBM10-silenced A549 cells by western blot analysis, normalized to GAPDH. *p < 0.05, **p < 0.01, ***p < 0.001. Data are means ± SD

RBM10 silencing resulted in decreasing PTEN protein level and increasing phospho‐PI3K, phospho‐AKT, and phospho-mTOR levels in NSCLC cells. Silencing Neat1_2 reversed these changes in expression in RBM10-silenced A549 cells (Fig. 7A–C). Together with the results presented above, these findings suggested that RBM10 promoted the expression of PTEN and suppressed the phosphorylation of PI3K, AKT, and mTOR via Neat1_2.

## Discussion

In recent years, several articles have reported the regulation of RBM10 in lung cancer [30, 33, 34]. Although there are conflicting conclusions about whether this protein promotes or inhibits lung cancer, most of the experimental results confirmed that RBM10 is a tumor suppressor factor [30, 34, 35]. We demonstrated by immunohistochemistry and western blot that RBM10 expression reduced in NSCLC tissues and cell lines. We subsequently investigated its effect on NSCLC phenotypes, and the results showed that RBM10 overexpression inhibited invasion and metastasis, whereas RBM10 silencing promoted these processes. The ability of tumor cells to invade and metastasize is enhanced through loss of epithelial features and acquisition of mesenchymal phenotypes in a process known as EMT [36]. EMT is often accompanied by upregulation of N-cadherin and Vimentin, and downregulation of E-cadherin. We found that RBM10 inhibited the expression of N-cadherin and Vimentin, and increased the expression of E-cadherin, further confirming that RBM10 inhibited invasion and metastasis of NSCLC.

In further investigation of the mechanism of RBM10 in NSCLC, Clip-Seq confirmed that RBM10 binds 4040 RNAs, including 353 lncRNAs. LncRNAs regulate gene expression not only at the post-transcriptional level but also at the transcriptional and epigenetic levels [37]. Therefore, the mechanism of RBM10 and LncRNA in NSCLC is the focus of our study. Four of the top ten loci in lncRNA according to maxHeight are Neat1, and we further confirmed through the RIP experiment that RBM10 can recruit Neat1. One study showed that Neat1 expression increased in LA tissues, and that ATF2 and Neat1 form a positive feedback loop mediated by miR-26a-5p, coordinately contributing to LA progression [38]. Zhao et al. concluded that downregulation of Neat1 in NSCLC inhibits its growth, migration, and invasion through the miR-204/NUAK1 axis [39]. Neat1 was further found to affect NSCLC by elevating its malignant potential via the miR-582-5p/EIF4G2 axis [40]. As we expected, RBM10 overexpression suppressed Neat1 expression, whereas RBM10 silencing promoted Neat1 expression.

From the structure of Neat1, we found two transcripts of Neat1 as Neat1_1 and Neat1_2. The latter is currently the only RNA molecule identified in paraspeckles, which are ribonucleoprotein bodies found in the interchromatin space of mammalian cell nuclei, coordinating various biochemical processes [23]. Overexpression of paraspeckles has been associated with some cancers and is often associated with poor prognosis [41, 42]. Two studies have shown that Neat1_2 promotes malignant biological behaviors in thyroid cancer through the competing endogenous RNA mechanism [43, 44]. The high expression of Neat1_2 in liver cancer is related to an increase in cisplatin resistance [45]. Knockdown of Neat1_2 increases the sensitivity of hepatocellular carcinoma cells to radiotherapy [46]. In contrast, Neat1_1 is not a key component of paraspeckles and has also been detected in several non-paraspeckle locations [47]. Carmen et al. showed that cancer cells lacking Neat1_1 did not exhibit cell cycle defects, and Neat1_1 specific knockout mice did not exhibit the phenotype observed in Neat1-deficient mice, suggesting that the function of Neat1 is mainly based on the Neat1_2 isoform [24]. And one study has shown that Neat1_2 has a stronger effect on RNA-RBP complex formation than Neat1_1 [48]. We demonstrated in RIP assay that RBM10 could recruit Neat1_2. Importantly, RBM10 overexpression significantly inhibited Neat1_2 expression, and RBM10 silencing significantly increased Neat1_2 expression. In addition, the proportion of Neat1_2 in Neat1 decreased significantly in RBM10 overexpressed A549, while the proportion of Neat1_2 in Neat1 increased significantly after RBM10 silencing. Based on the ratio of Neat1_2 in Neat1 and the expression of Neat1 and Neat1_2, we concluded that Neat1_1 increased after RBM10 overexpression and decreased after RBM10 silencing.

RBM10 is a kind of AS factor that is enriched in the splicing sites at the 5′ and 3′ ends of introns and exons of pre-mRNA, with more abundant binding near the 3′ splicing site than those of the 5′ splicing site. By binding to small nuclear ribonucleoprotein and cleavage sites of pre-mRNA substrates, RBM10 can synergistically remove introns and increase exon jumping events by more than 74% [49]. The change in abundance and activity of RBM10 can lead to changes in some gene splicing patterns. Nicotine is a known risk factor for the development of lung cancer. A transcriptome sequencing study demonstrated that compared with the epithelial cells of normal controls, those of the nicotine exposure group showed a significant decrease in the expression level of Neat1, which was a result of AS [50]. Our data suggest that RBM10 regulates AS of Neat1, since RBM10 could upregulate Neat1_1 and downregulate Neat1_2. Also, we observed that RBM10 can also affect Neat1 through other mechanisms, because RBM10 inhibited total NEAT1 expression.

In addition, previous studies have shown that RBM10 regulates the phosphorylation of the RAP1/AKT/CREB [30] and MAPK/PI3K/AKT [31] signaling pathways in lung cancer. The PI3K/AKT/mTOR signaling axis may be a key molecular pathway in lung tumorigenesis [51–54]. Accordingly, inhibitors of PI3K/AKT/mTOR signaling have been suggested as potential therapeutic agents for NSCLC [55–57]. PTEN is a tumor suppressor gene with phosphatase activity, which can inhibit the development of tumors by antagonizing the activity of phosphorylase, such as tyrosine kinase. PTEN inhibits the PI3K signaling pathway by dephosphorylating phosphatidylinositol-3,4,5-triphosphate to phosphatidylinositol-4,5-bisphosphate [58]. It has been reported that Neat1 promotes tumor development by inhibiting PTEN expression in laryngeal [28] and cervical cancer [59]. We investigated the relationship among RBM10, Neat1_2, and PTEN and the phosphorylation of downstream genes. We found that RBM10 promoted the expression of PTEN by inhibiting Neat1_2, which in turn inhibited the phosphorylation of the PI3K/AKT/mTOR signaling pathway.

## Conclusions

**Fig. 8:**
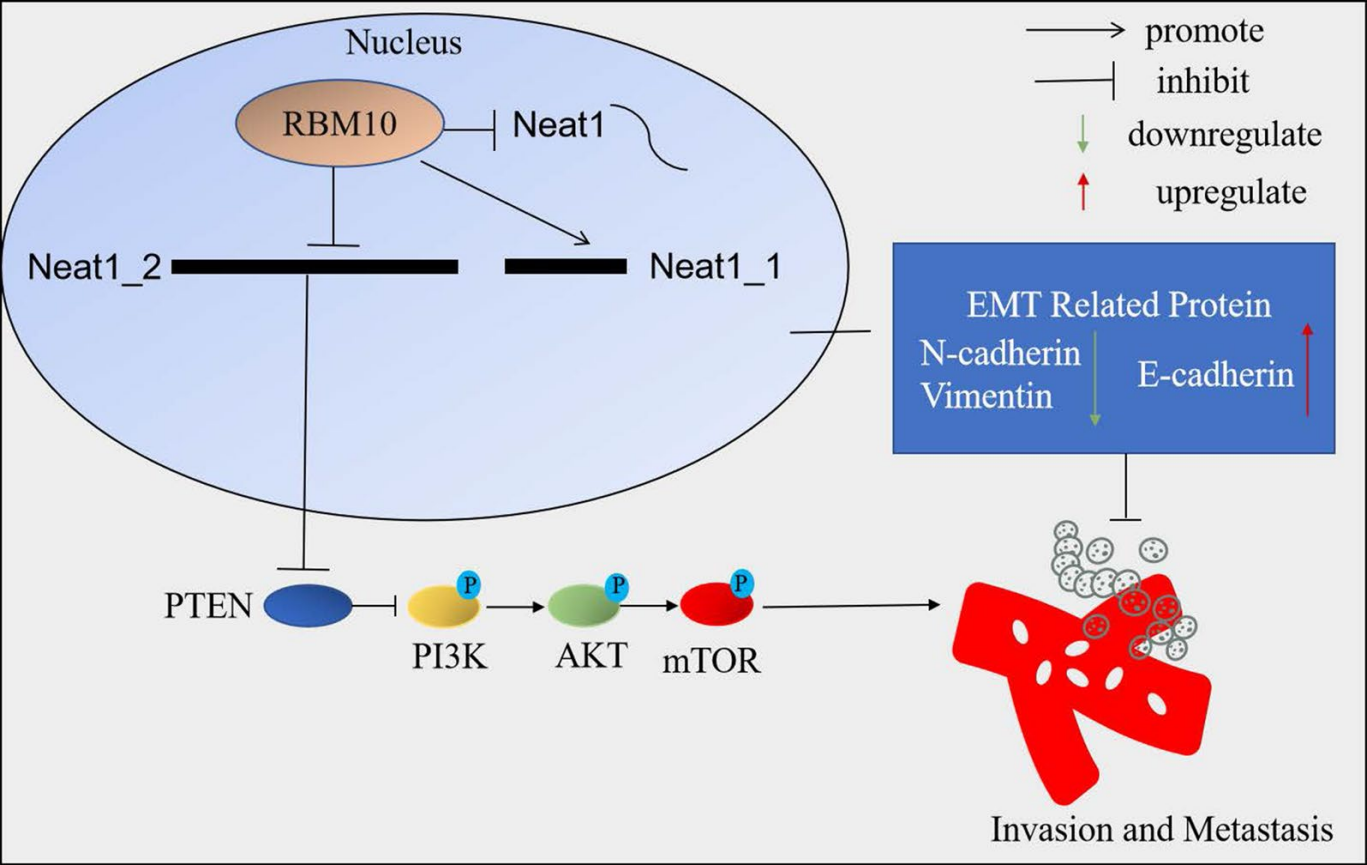
Mechanism of RBM10 and Neat1 in invasion and metastasis of NSCLC.RBM10 regulates AS of Neat1, leading to changes in the expression level of Neat1_2 and Neat1_1. RBM10 decreases Neat1_2 to inhibit the invasion and metastasis of NSCLC via PTEN/PI3K/AKT/mTOR signaling

In summary, RBM10 regulates AS of Neat1, leading to changes in the expression level of Neat1_2 and Neat1_1. RBM10 decreases Neat1_2 to inhibit the invasion and metastasis of NSCLC via PTEN/PI3K/AKT/mTOR signaling. The oncogenic effect of Neat1 is mainly attributed to Neat1_2. These data on multiple relationships of RBM10 and Neat1 in NSCLC contribute to our understanding of the detailed molecular mechanisms involved in the progression of NSCLC (Fig. 8).

## Supplementary Information

**Additional file 1: Figure S1.** The flow chart of Clip-Seq.

## Data Availability

All data generated or analyzed during this study are included in this published article and its supplementary information files.

## Abbreviations

NSCLC: Non-small cell lung cancer
RBP: RNA-binding protein
NcRNA: Non-coding RNA
RBM10: RNA-binding motif protein 10
LA: Lung adenocarcinoma
AS: Alternative splicing
Neat1: Nuclear enriched abundant transcript 1
LncRNA: Long non-coding RNA
Clip-Seq: Crosslinking-immunprecipitation and high-throughput sequencing
IOD: Integral optical density
SD: Standard deviation
SCC: Squamous cell carcinoma
PBS: Phosphate buffer solution
RT-qPCR: Reverse transcription quantitative polymerase chain reaction
RIP: RNA immunoprecipitation
EMT: Epithelial-mesenchymal transition
