# Supplementary figures and images for: RBM10 regulates alternative splicing of lncRNA Neat1 to inhibit the invasion and metastasis of NSCLC
Source: Cancer Cell Int. 2022 Nov 5;22:338. doi: 10.1186/s12935-022-02758-w (PMC9636673; doi:10.1186/s12935-022-02758-w)

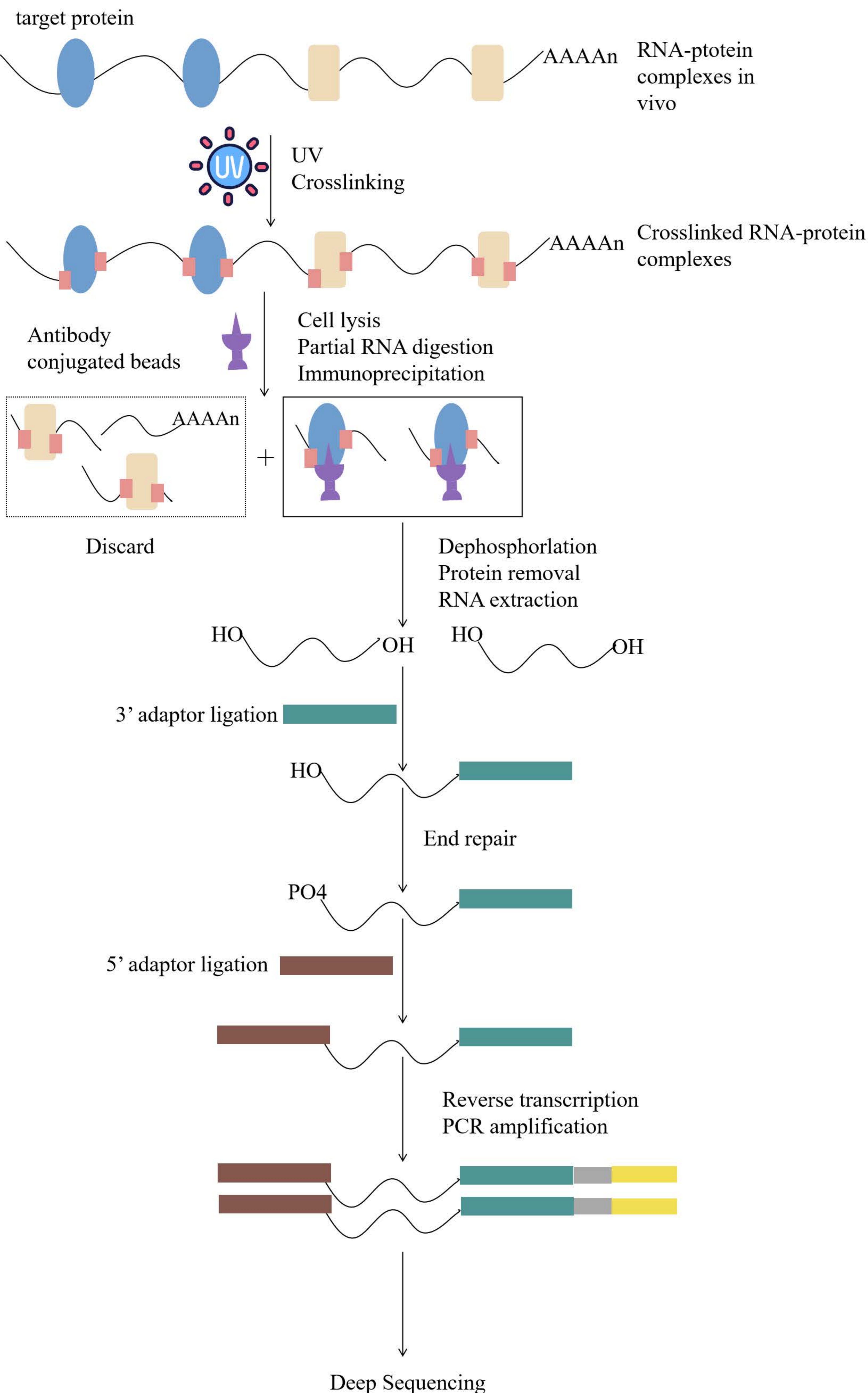

Supplement: Supplementary file 1 — Additional file 1: Figure S1. The flow chart of Clip-Seq. [file 12935_2022_2758_MOESM1_ESM.pdf]
